# A research algorithm to improve detection of delirium in the intensive care unit

**DOI:** 10.1186/cc5027

**Published:** 2006-08-18

**Authors:** Margaret A Pisani, Katy LB Araujo, Peter H Van Ness, Ying Zhang, E Wesley Ely, Sharon K Inouye

**Affiliations:** 1 Department of Internal Medicine, Pulmonary & Critical Care Section, and the Program on Aging, Yale University School of Medicine, Cedar Street, New Haven, Connecticut 06520-8057, USA; 2 Department of Internal Medicine, Geriatrics Section, and the Program on Aging, Yale University School of Medicine, Cedar Street, New Haven, Connecticut 06520-8057, USA; 3 Department of Medicine and Center for Health Services Research, Veterans Affairs Geriatric Research and Clinical Education Center (GRECC) and the Vanderbilt University School of Medicine, 6109 Medical Center East, Nashville, Tennessee 37232, USA; 4 Department of Medicine, Beth Israel Deaconess Medical Center, Harvard Medical School and the Aging Brain Center, Hebrew Rehabilitation Center for Aged, 1200 Centre Street, Boston, Massachusetts 02131, USA

## Abstract

**Introduction:**

Delirium is a serious and prevalent problem in intensive care units (ICUs). The purpose of this study was to develop a research algorithm to enhance detection of delirium in critically ill ICU patients using chart review to complement a validated clinical delirium instrument.

**Methods:**

A prospective cohort study was conducted in 178 patients aged 60 years and older who were admitted to the medical ICU. The Confusion Assessment Method for the ICU (CAM-ICU) and a validated chart review method for detecting delirium were performed daily. We assessed the diagnostic accuracy of the chart-based delirium method using the CAM-ICU as the 'gold standard'. We then used an algorithm to detect delirium first using the CAM-ICU ratings and then chart review when the CAM-ICU was unavailable.

**Results:**

When using both the CAM-ICU and the chart-based review, the prevalence of delirium was found to be 80% of patients (143 out of 178) or 64% of patient-days (929 out of 1,457). Of these patient-days, 292 were classified as delirium by the CAM-ICU. The remainder (637 patient-days) were classified as delirium by the validated chart review method when CAM-ICU was missing because the assessment was conducted for weekends or holidays (404 patient-days), when CAM-ICU was not performed because of stupor or coma (205 patient-days), and when the CAM-ICU was negative (28 patient-days). Sensitivity of the chart-based method was 64% and specificity was 85%. Overall agreement between chart and the CAM-ICU was 72%.

**Conclusion:**

Eight out of 10 patients in this cohort study developed delirium in the ICU. Although use of a validated delirium instrument with frequent direct observations is recommended for clinical care, this approach may not always be feasible, especially in a research setting. The algorithm proposed here comprises a more comprehensive method for detecting delirium in a research setting, taking into account the fluctuation that occurs with delirium, which is a key component of accurate determination of delirium status. Improving detection of delirium is of paramount importance both to advance delirium research and to enhance clinical care and patient safety.

## Introduction

Delirium is a common disorder among older intensive care unit (ICU) patients because of their advanced age, critical illness, and multiple medical procedures and interventions [1-3]. Mechanically ventilated patients are at risk for the development of delirium due to multi-system illnesses, co-morbidities, and medications. In the ICU, delirium negatively affects 6-month survival and weaning from mechanical ventilation, and contributes to the development of nosocomial pneumonia and increased length of stay [4-6]. Delirium has also been associated with higher hospital and ICU costs, which appear to increase linearly with severity of delirium [7].

By definition, delirium is an acute disorder of attention and global cognitive function, characterized by acute onset and fluctuating symptoms. The critical nature of underlying illnesses and lack of verbal communication in ICU patients renders delirium assessment in the ICU particularly difficult. The Society for Critical Care Medicine sedation guidelines [8] recommend delirium assessment in all ICU patients using a validated assessment instrument. Recent studies have documented the usefulness of the Confusion Assessment Method for the ICU (CAM-ICU) in detecting delirium in critically ill patients [3,9,10]. The CAM-ICU and the Intensive Care Delirium Screening Checklist [11] are the current standard instruments for detecting delirium in the ICU. In the absence of such assessments, however, delirium in the ICU is frequently missed because of its predominately hypoactive state [12,13].

The majority of research studies conducted to date in the ICU have measured delirium at one point in time during a 24-hour period. Given the fluctuating nature of delirium, this approach limits the sensitivity of delirium detection by potentially missing delirium that occurs before or after the delirium assessment, which in turn can result in underestimation of the overall prevalence of delirium [14]. Two recent publications [15,16] evaluated delirium more than once a day in the ICU. The aim of this report is to describe our research method for detecting delirium in the ICU. This method, which utilizes a validated observation-based delirium instrument administered once daily combined with a validated chart review method, allows us to address better the acute onset and fluctuating nature of delirium and enhances detection of delirium.

## Materials and methods

### Study participants

The study participants were 178 patients aged 60 years or older who were admitted to the medical ICU at Yale-New Haven Hospital from 3 September 2002 through to 30 September 2003. Yale-New Haven Hospital is an 800-bed urban teaching hospital with a 14-bed medical ICU. Age-eligible patients were excluded from the study if there was no identifiable proxy to provide information about the patient, if they expired before the proxy interview could be obtained, if they were transferred from another ICU because of missing baseline data, if they were admitted to the medical ICU for less than 24 hours, or if they were non-English speaking. Of the 396 patients screened, 183 were eligible for enrollment. The numbers of patients who were not eligible for inclusion were as follows: 30 had no identifiable proxy; 11 were non-English speaking; 30 were unable to communicate before ICU admission (for instance, because of aphasia or total deafness); 100 were admitted to the ICU for less than 24 hours; and 42 were transferred from another ICU. Of the 183 eligible patients, 178 (97%) were enrolled in the study. Five eligible patients were excluded because of proxy refusal. Informed consent for participation was obtained from the proxy respondents according to procedures approved by the Institutional Review Board of Yale University School of Medicine. When possible, assent was also obtained from patients.

### Patient interviews

Delirium was assessed by trained research nurses from Monday to Friday using the CAM-ICU [2,3]. The CAM-ICU, an adapted version of the Confusion Assessment Method [17], consists of a brief interview with the patient and incorporates the Diagnostic and Statistical Manual III-R operationalized criteria to define delirium. It is currently the most widely used method for assessing delirium in critically ill patients. Delirium assessment using the CAM-ICU incorporates four key features that constitute the definition of delirium, as taken from the original Confusion Assessment Method algorithm presented by Inouye and coworkers [17]. The instrument includes a series of nonverbal tasks to rate the four key criteria: acute change from baseline or fluctuating course, inattention, disorganized thinking, and altered level of consciousness. All tasks and questions were designed to be completed by nonverbal, mechanically ventilated, or restrained patients in ICU settings. The CAM-ICU was validated in three large cohort studies of ICU patients against delirium expert assessments, and was found to have a sensitivity of 95–100%, a specificity of 89–93%, and high interobserver reliability [2,3,10].

The Richmond Agitation-Sedation Scale (RASS) [18,19] was used to assess sedation status. The RASS is a ten-point rating scale with four levels for agitation, five levels for sedation, and one level for calm, awake patients. This scale was designed with the anchor centered at level 0, positive ratings for agitation, and negative ratings for sedation. It completely separates ratings according to a patient's responses to verbal and then to physical stimulation. This sedation scale has excellent inter-rater reliability and has been validated for criterion and construct validity [18]. The CAM-ICU is not performed in patients who are not arousable (stupor: RASS of -4; coma: RASS of -5); when patients had a RASS of -4 or -5 or were unavailable for interview, two more attempts were made during the day to interview the patient.

Inter-rater agreement for the CAM-ICU was 100% between the two research nurse interviewers for this study. All questionnaires were pilot tested before the beginning of the study, and all research nurse interviewers were trained and standardized in the interview process.

### Chart review

Daily chart review during the ICU stay was conducted to detect evidence of delirium during the previous 24 hours. We used a previously validated chart review method to detect delirium [20]. The whole medical record was reviewed, including but not limited to progress notes, nursing notes, and consult notes. A geriatric research nurse, who underwent extensive training in the chart-based delirium detection method, conducted the medical record abstractions. The ICU nurses caring for the patients were unaware of the study hypothesis or the CAM-ICU ratings performed by the research nurse. The abstractor coded delirium as 'yes' if any key terms or descriptors were present and evidence of acute onset or fluctuation in symptoms was present. Specifically, the abstractor considered the following question: 'Is there any evidence from the chart of acute confusional state (for example, delirium, mental status change, inattention, disorientation, hallucinations, agitation, inappropriate behavior, or other)?' This chart abstraction method for delirium detection has previously been shown to have a sensitivity of 74% and a specificity of 83% in a non-ICU population [20]. If delirium was coded as 'yes', then the abstractor recorded the sources of information (nurse, physician, or other) and the nursing shift or shifts on which delirium was noted. The medical record review required between 15 and 30 min per patient.

We compared the chart-based identification of patient-days of delirium with the research nurse rating using the CAM-ICU (reference standard). We calculated overall agreement, sensitivity, specificity, false-positive rate, false-negative rate, positive predictive value, negative predictive value, and their related 95% confidence intervals.

### Sedation assessment

The research nurse conducted a sedation assessment using the RASS each time the CAM-ICU rating was completed. In addition, the ICU nurses record the RASS every hour as part of clinical care; the worst RASS recorded was abstracted from the medical record throughout each 24-hour period. Fluctuation in sedation status was determined using the RASS assessments and chart review for sedation level (for example, unresponsive, agitated, lethargic, or alert). Fluctuation was defined as at least two changes between categories during a 24-hour period.

### Delirium detection

Delirium status was determined each day of the ICU stay based on the following hierarchy. Priority was assigned to the rating determined by the CAM-ICU, completed via direct observation by the study nurse. If the CAM-ICU was positive for delirium, then the patient was recorded as delirious for the day. If the CAM-ICU was negative or the patient interview was not conducted because a research nurse was not available, or if the patient had a RASS of -4 or -5 at the time of nurse evaluation, then we turned to the medical record for evidence of delirium. If the medical record revealed evidence of delirium on that date, then the patient was recorded as delirious.

## Results

Baseline characteristics of the study population are presented in Table 1. The age (mean ± standard deviation) of the participants was 74.2 ± 8.3 years, half of them were women, and 85% were admitted from home. The majority of admissions were for a respiratory diagnosis, and 58% required mechanical ventilation. The length of ICU stay (mean ± standard deviation) as 8.2 ± 9.3 days, and the median length of ICU stay was 5.0 (range 1–51) days.

One hundred and forty-three participants (80%) had delirium at some point during their ICU stay. The 178 participants had 1457 daily assessments during their ICU stay. All analyses in this study are presented as patient-days. Of 1457 patient-days, 929 (64%) were classified as delirious.

As shown in Table 2, 187 of the 292 patient-days rated as delirious by the CAM-ICU (reference standard) were correctly identified using the chart-based delirium instrument, giving a 64% sensitivity and a 36% false-negative rate. The chart delirium rating indicated no delirium in 156 out of 184 patients rated as not delirious by the CAM-ICU, giving a specificity of 85% and a 15% false-positive rate. Although the positive predictive accuracy of 87% indicates that a positive result on the chart instrument is helpful in detecting delirium, the 60% negative predictive accuracy suggest that the absence of chart documentation cannot reliably exclude delirium in an ICU population.

The CAM-ICU was not performed on 703 (48%) patient-days, for the reasons presented in Table 3. The majority of these missing CAM-ICU ratings (76% [533/703]) were because the assessment was conducted for weekends or holidays when research staff was unavailable. Table 4 presents our chart review for delirium when the CAM-ICU was not performed. Of the 278 patient-days when the CAM-ICU was not performed because of the presence of stupor or coma at the time of interview, 205 (74%) had chart evidence of delirium and 73 (26%) had no chart evidence of delirium. Of the 703 patient-days on which the CAM-ICU was not attempted, chart review identified 404 (58%) patient-days of delirium. Using both CAM-ICU and chart review, delirium detection improved from 292 delirium days out of 1,457 (20%) by CAM-ICU alone to 929 delirium days (64%) using the algorithm proposed in the present study.

Table 5 presents chart review documentation of delirium by practitioner and nursing shift. Of the 824 patient-days for which there was chart documentation of delirium, 710 (86%) instances were noted in the nursing notes, 392 (47%) in physician notes, and 272 (33%) in both nursing and physician notes. Delirium was most often documented in the chart on the day shift (08:00 hours to 16:00 hours), with 580 (70%) of patient-days. Forty-nine per cent of the time (402 patient-days), delirium was documented in the chart on multiple nursing shifts.

## Discussion

We present a useful research algorithm for detecting delirium in an ICU setting. This method utilizing both the CAM-ICU and a validated chart review demonstrates a more comprehensive approach to detection of delirium for research purposes.

Compared with research nurse ratings using the CAM-ICU, the chart-based method has a sensitivity of 64% and a specificity of 85%. The positive predictive accuracy of the chart-based method was 87%, which is much higher than the 39% reported in a non-ICU population and is probably related to the greater prevalence of delirium in the ICU [20].

Numerous studies have verified the under-recognition and under-documentation of delirium by both physician and nursing staff [21-23]. Under-documentation of delirium in the medical record is supported by our findings, in that there was no chart documentation for 36% (105/292) of delirium cases identified by the CAM-ICU.

Our false-positive rate for chart-based detection was 15%. Because of the fluctuating nature of delirium, the CAM-ICU may miss cases of delirium if it is performed only once a day. In this study research nurses performed the CAM-ICU during the day shift. When we examined shift of chart documentation for our 28 false-positive patients, we found that only five had chart documentation on the day shift whereas the rest were documented on nights only or evenings only or some combination of shifts. This reflects the fluctuating nature of delirium. In addition, our findings on the prevalence of delirium (80%) are similar to those other studies that reported on ICU delirium (40–87%) [1-3,24,25].

Delirium is a fluctuating disorder, and reliance on a single daily observation can substantially underestimate the prevalence of delirium. Given the false-negative rate of 36% for chart review, we recommend that the CAM-ICU be used as the primary tool for detecting delirium in the ICU. For clinical care, ICU nurses should be trained to administer the instrument on each shift concurrent with assessments of sedation and acuity. Ely and coworkers [15,26] have demonstrated that large-scale implementation of the CAM-ICU by nursing staff is feasible. However, when frequent CAM-ICU assessment is not feasible or when research staff members are unavailable, such as during weekends or holidays, using a validated chart review will markedly improve detection of delirium in the ICU, from 20% to 64% patient-days in our study. Chart review for detection of delirium has been used in multiple studies [20,27]. Use of both the CAM-ICU and chart review represents a comprehensive delirium detection method in the ICU.

Depending on the nature of the study, coma and stupor may or may not have been included in delirium rates in previous studies [1,6,28,29]. Prior research [30,31] suggested that there is a spectrum of abnormal mental state and that patients may move between delirium, stupor, and coma. The CAM-ICU cannot be performed when a patient is in a state of stupor or coma, and these patients are often excluded from analysis when delirium and its impact on outcomes are evaluated. Previous research suggests that a large number of patients who have coma or stupor transition to delirium in the ICU. McNicoll and coworkers [1] reported that 85% of patients who had coma or stupor transitioned to delirium, whereas 12% remained in coma/stupor and 3% transitioned to no delirium. In our study, of the 278 patient-days on which the CAM-ICU could not be performed because of stupor or coma, 205 (74%) had chart evidence for delirium. Only 5% (73/1,457) of our patient-days were stupor/coma with no chart documentation of delirium, and these were not counted as delirium but rather handled as a separate categorization.

Not surprisingly, the nursing staff documented delirium in their notes more frequently than did the physicians. The nurse-to-patient ratio in our ICU is usually 1:2, and thus the nurses spend much more time with the patients over the course of the day, allowing them to note changes in mental status as well as fluctuation in mental status. Our ICU nurses also give detailed sign out information when changing shifts so that the next nurses on duty are aware of each patient's baseline mental status, allowing them to assess better any changes that subsequently occur.

The strengths of the present study include the sizeable nature of the patient group with detailed daily clinical observations on 1,457 patient-days by a skilled and highly reliable research team. We applied two well validated instruments for delirium detection. In addition, this prospective ICU cohort was representative of the medical ICU population at our hospital. However, several caveats about the study deserve comment. No 'gold standard' method was used to validate our delirium diagnoses; however, both delirium measures used have been externally validated and are widely employed. One research nurse performed the chart-based abstraction, and this may be a potential source of bias and limit the generalizability of the findings. As with any single-site study, the generalizability of the results may be called into question. Although the external validity could be challenged, this does not compromise the internal validity of our findings, which require replication in other settings and populations. Finally, the proposed algorithm is intended for use in research studies and not for general clinical purposes, where more frequent application of the CAM-ICU is recommended because of its superior performance compared with the chart review method.

## Conclusion

Delirium has a high prevalence in the older critically ill population [1,3], where increasing age and cognitive impairment represent important risk factors. Delirium has been shown to have impacts on both short-term and long-term outcomes from both ICU and hospital care [4,6,7,32-34]. As studies move forward to improve our understanding of modifiable risk factors for delirium and ultimately to assist in its prevention and treatment, it will be of critical importance to rate correctly a patient's delirium status during the ICU stay. Augmenting delirium instruments with multiple sources, such as the medical chart, is a method that has previously been applied in non-ICU studies [27] and is probably even more important in the ICU setting. Thus, the algorithm presented here using a combination of CAM-ICU and chart review will aid researchers undertaking studies of delirium to better identify this high-risk condition and intervene.

## Key messages

• The CAM-ICU should be used to clinically screen patients for delirium and should ideally be performed at least once per nursing shift.

• Screening for delirium in research studies should include both the CAM-ICU and chart review due to delirium's fluctuating nature.

• While the presence of chart documentation has a good positive predictive value for detecting delirium, the absence of chart documentation cannot reliably exclude delirium in an ICU population.

## Abbreviations

ICU = intensive care unit; CAM-ICU = Confusion Assessment Method for the Intensive Care Unit; RASS = Richmond Agitation-Sedation Scale.

## Competing interests

Dr Pisani is a recipient of a NIH K23 Mentored Career Development Award (K23 AG 23023-01A1). Dr Inouye is supported in part by grants #R21AG025193 and #K24AG000949 from the National Institute on Aging. Dr Ely is a recipient of an NIH K23 award from the NIA (K23 AG01 023-01A1). None of the authors has a financial or other potential conflict of interest.

## Authors' contributions

MP conceived and designed the study, interpreted the data, and drafted the manuscript. KA designed the study, conducted data acquisition and analysis, and manuscript revision. PV analyzed and interpreted data, and conducted manuscript revision. YZ designed the design and conducted data analysis. WE interpreted data and revised the of manuscript. SI designed the study, interpreted data, and revised the manuscript. All authors read and approved the final manuscript.

## Figures and Tables

**Table 1:** Baseline characteristics

Variable	Value
Characteristic	
Age (years; mean ± SD)	74.2 ± 8.3
Male sex (*n *[%])	92 (52)
Non-white race (*n *[%])	22 (12)
Medicaid status (*n *[%])	24 (14)
Admitted from nursing home (*n *[%])	26 (15)
Currently married (*n *[%])	95 (53)
Health measures	
Dementia, n (%)	52 (29)
Any disability in activities of daily living (*n *[%])	55 (31)
Any disability in independent activities of daily living (*n *[%])	151 (85)
APACHE II score (mean ± SD)	23.4 ± 6.3
Charlson Comorbidity Index (mean ± SD)	1.9 ± 2
Full code status on admission (*n *[%])	150 (84)
Intubation (*n *[%])	104 (58)
Non-invasive ventilation (*n *[%])	47 (26)
Pulmonary artery catheter placement (*n *[%])	19 (11)
Hemodialysis (*n *[%])	9 (5)
Length of ICU stay (days; mean ± SD)	8.2 ± 9.3
Length of ICU stay (days; median [range])	5 (1–51)
Admitting diagnosis	
Gastrointestinal hemorrhage (*n *[%])	29 (16)
Respiratory (*n *[%])	91 (51)
Neurologic (*n *[%])	4 (2)
Sepsis (*n *[%])	31 (17)
Other (*n *[%])	23 (13)

A total of 178 patients were included in the study. APACHE, Acute Physiology and Chronic Health Evaluation; ICU, intensive care unit; SD, standard deviation.

**Table 2:** Performance of chart-based delirium detection compared with the CAM-ICU by patient-days

Delirium from chart	Delirium from CAM-ICU		Total
		
	Yes	No	
Yes	187^a^	28^b^	215
No	105^c^	156^d^	261
			
Total	292	184	476

^a^83 participants, ^b^26 participants, ^c^58 participants, ^d^83 participants. Overall agreement: 72% (95% CI 68–76%). Sensitivity: 64% (95% CI 59–70%). Specificity: 85% (95% CI 80–90%). False-negative rate: 36% (95% CI 30–41%). False-positive rate: 15% (95% CI 10–20%). Positive-predictive accuracy: 87% (95% CI 82–91%). Negative-predictive accuracy: 60% (95 CI 54–66%). Confusion Assessment Method for the Intensive Care Unit; CI, confidence interval.

**Table 3:** Reason CAM-ICU not performed by patient-days

Reason	*n *(%)
Non-interview days (holidays, weekends)	533 (76%)
Not available (not in room, tests)	18 (3%)
Lethargica	95 (14%)
Alert but unresponsive to interviewer^a^	2 (<1%)
Agitated^a^	31 (4%)
Terminal care	6 (1%)
Other (for instance, nurse requested patient not be interviewed, discharged early)	9 (1%)
Patient or surrogate refusal	9 (1%)

A total of 703 patient-days were included in this analysis. ^a^The 128 (18%) of cases for which the CAM-ICU could not be completed by the research nurse due to lethargy, alert but un-responsive, and agitated were probably manifestations of delirium. Confusion Assessment Method for the Intensive Care Unit.

**Table 4:** Chart-based review for delirium when the CAM-ICU was not performed by patient-days

Reason why CAM-ICU results not available	Chart evidence of delirium (patient-days)	No chart evidence of delirium (patient-days)	Total
Coma/stupor at time of CAM-ICU	205 (74%)^a^	73 (26%)^b^	278
CAM-ICU not performed	404 (58%)^c^	296 (42%)^d^	700^e^
Total	609 (62%)	369 (38%)	978^e^

^a^66 participants, ^b^33 participants, ^c^115 participants, ^d^125 participants, ^e^chart data missing in three cases. Confusion Assessment Method for the Intensive Care Unit.

**Table 5:** Chart review documentation of delirium by practitioner and nursing shift by patient-days

Practioner reporting and nursing shift	Chart review documentation of delirium
Practioner reporting	
Physicians	392
Nurses	710
Physicians and nurses	272
Other^a^	4
Nursing shift	
Days (08:00 hours to 16:00 hours)	580
Evenings (16:00 hours to 00:00 hours)	374
Nights (00:00 hours to 08:00 hours)	371
Multiple shifts	402

A total of 824 patient-days were included in the analysis. ^a^Other includes dietician (*n *= 1), social worker (*n *= 2), and family (*n *= 1).
